# Astragalus saponins downregulate vascular endothelial growth factor under cobalt chloride-stimulated hypoxia in colon cancer cells

**DOI:** 10.1186/1472-6882-12-160

**Published:** 2012-09-19

**Authors:** Pui-Ching Law, Kathy K Auyeung, Lok-Yi Chan, Joshua K Ko

**Affiliations:** 1 Center for Cancer and Inflammation Research, School of Chinese Medicine, Hong Kong Baptist University, Hong Kong SAR, China

**Keywords:** Astragalus saponins, Hypoxia, VEGF, Akt/mTOR, COX-2, Colon cancer

## Abstract

**Background:**

Our ongoing research has revealed that total saponins extracted from the medicinal herb Radix Astragali (AST) exhibits significant growth-inhibitory and proapoptotic effects in human cancer cells. In the present study, the potential of AST in controlling angiogenesis was further investigated with elaboration of the underlying molecular mechanism in human colon cancer cell and tumor xenograft.

**Results:**

AST decreased the protein level of VEGF and bFGF in HCT 116 colon cancer cells in a time- and dose-dependent manner. Among the Akt/mTOR signal transduction molecules being examined, AST caused PTEN upregulation, reduction in Akt phosphorylation and subsequent activation of mTOR. AST also suppressed the induction of HIF-1α and VEGF under CoCl_2_-mimicked hypoxia. These effects were intensified by combined treatment of AST with the mTOR inhibitor rapamycin. Despite this, our data also indicate that AST could attenuate cobalt chloride-evoked COX-2 activation, while such effect on COX-2 and its downstream target VEGF was intensified when indomethacin was concurrently treated. The anti-carcinogenic action of AST was further illustrated in HCT 116 xenografted athymic nude mice. AST significantly suppressed tumor growth and reduced serum VEGF level *in vivo*. In the tumor tissues excised from AST-treated animals, protein level of p-Akt, p-mTOR, VEGF, VEGFR1 and VEGFR2 was down-regulated. Immunohistochemistry has also revealed that AST effectively reduced the level of COX-2 in tumor sections when compared with that in untreated control.

**Conclusion:**

Taken together, these findings suggest that AST exerts anti-carcinogenic activity in colon cancer cells through modulation of mTOR signaling and downregulation of COX-2, which together reduce VEGF level in tumor cells that could potentially suppress angiogenesis.

## Background

Epidemiological studies have suggested that herbal medicines and fruit extracts play a major role in the prevention and treatment of many types of cancer including that of the colon [1-3]. Radix Astragali is frequently used as health food supplement in Asian population and also serves as a lead herb in many Traditional Chinese Medicine formulations. Total *Astragalus* saponins (AST) are the major active constituent found in this herb and its anti-cancer effects have been investigated for some times. Results from our previous investigations demonstrated that AST could exert cell growth inhibition in various cancer cell lines through regulation of cell proliferation and apoptosis [4,5]. AST also possesses prominent effects against colon cancer growth in HT-29 nude mice tumor xenograft with much fewer adverse effects compared to conventional chemotherapeutic drugs [5]. Recently, we found that AST could also reduce cell invasiveness and angiogenesis in gastric cancer cells [6]. In this study, we attempted to explore the possible anti-angiogenic effects of AST in colon cancer and to unveil the underlying mechanism.

Angiogenesis is essential for the initiation, progression and metastasis of solid tumor. Overexpression of angiogenic factors can direct the endothelial cell proliferation and sprouting in tumor mass as well as maintain vascular state of the tumor for the growth [7]. Vascular endothelial growth factor (VEGF) has been identified as the most important angiogenic factor for tumor progression because it is released by a variety of tumor cells and overexpresses in different human cancers. Drugs that can inhibit the production of VEGF or block its receptor signaling show significant inhibition of tumor growth [8-10]. Bevacizumab, a recombinant human monoclonal antibody directed against VEGF, has shown promising effects when used as combination therapy in advanced colorectal cancer patients [11].

Intra-tumoral hypoxia is a common phenomenon as the rapid growing cells deplete oxygen in the cellular microenvironment. A series of adaptive responses will be triggered, which involves the elevation of the transcription and subsequent translation of genes responsible for cell survival, glucose metabolism, angiogenesis and invasion [12]. Activation of hypoxia-inducible factor-1 alpha (HIF-1α) plays a major role in the development of tumor phenotype, especially in aggressive tumors [13]. Induction of VEGF expression promotes angiogenesis, which is mediated primarily through HIF-1α [14,15]. Under hypoxic condition, the ubiquitination of HIF-1α is inhibited and its accumulation transcriptionally activates *VEGF* gene by binding to a hypoxia responsive element (HRE) of the VEGF promoter [12]. Development of drugs targeting on the HIF system and VEGF is currently under active investigation in order to establish a target-oriented cancer therapy [16].

Cyclooxygenase-2 (COX-2), which is originally found to be an inflammatory mediator and a key rate-limiting enzyme in prostaglandins (PGs) production, is overexpressed at multiple stages of colon carcinogenesis. The role of COX-2 in tumor angiogenesis has been established since emerging evidence showed that inhibition of this pathway reduced tumor growth by suppressing VEGF expression and formation of blood vessels [17]. It was also found that *COX-2* is a direct target gene of HIF-1 in colon cancer cells. The overexpression of COX-2 in physical-stimulated or chemical-induced hypoxia enhanced VEGF production, which was accompanied by upregulation of PGE_2_ level in several human cancer cell lines [18,19]. NSAID, either COX-2 selective or nonselective, can block angiogenesis induced by co-cultured colon cancer cells [20]. The phosphatidylinositol 3’-kinase p85α (*PI3K*) gene is an oncogene in human ovarian and colon tumors [21]. PI3K/Akt/mTOR cascade is one of the growth factor-dependent signaling pathways modulating the amplitude of HIF-1α activation [22,23]. PI3K phosphorylates phosphatidylinositol-4,5-bis- phosphate (PIP2) to form phosphatidylinositol-4,5-triphosphate (PIP3), and such conversion can be blocked by the tumor suppressor gene PTEN. Activated PI3K recruits downstream molecule Akt, followed by activation of the mammalian target of rapamycin (mTOR). Apart from regulating cell growth and development [24], mTOR signaling has also attributed to angiogenesis through the induction of VEGF along with its receptor, both mediated by the increase of HIF-1α under hypoxic condition [25]. Interestingly, mTOR pathway was involved in the suppression of cell growth by COX-2 inhibitor in colorectal cancer [26], and the anti-angiogenic effect of mTOR inhibitor was in turn mediated by COX-2 in Ewing sarcoma [27].

The present study aimed at investigating the potential anti-angiogenic effect of AST in colon cancer, by examining the modulation of hypoxia-inducible HIF-1α and VEGF and the involving signaling pathways.

## Methods

### Extraction of Astragalus saponins (AST) and induction of hypoxia

Radix *Astragalus membranaceus* (Fisch.) Bunge var. *mongolicus* was obtained from the province of Shanxi, China. Total *Astragalus* saponins extract was prepared as described previously [5]. In brief, the herb was refluxed with 2% potassium hydroxide in methanol for 1 h. The solvent was evaporated and reconstituted with water. Butan-1-ol was then added for phase separation. Total saponins (AST) obtained were lyophilized into dry powder (about 0.6% w/w) and dissolved in ultrapure water to form a 10 mg/ml stock. To mimic a hypoxic condition, cells were treated with 100 μM cobalt chloride (CoCl_2_) 30 min prior to various drug treatments. The concentrations of AST being used in the *in vitro* study were chosen based on our findings from previous studies [4].

### Cell culture

Human colon adenocarcinoma cell lines HCT 116 and HT-29 were obtained from American Type Culture Collection (Manassa, VA) and cultured in Dulbecco’s Modified Essential Medium (DMEM) supplemented with 10% fetal bovine serum (FBS) plus 1% penicillin and streptomycin. Cultures were maintained in a humidified incubator with 5% CO_2_ at 37°C. All chemicals used for cell culture were purchased from Gibco (Carlsbad, CA).

### Antibodies and reagents

Rapamycin and LY 294002 were purchased from Merck (Whitehouse Station, NJ). Sources of antibodies are as follows: bFGF (BD Pharmingen, San Jose, CA); p-Akt (Ser 473), Akt, p-JNK, total JNK, p-mTOR, mTOR, p-p38 and total p38 (Cell Signaling Technology, Danvers, MA); β-actin (Sigma-Aldrich, St Louis, MO); HIF-1α, PTEN, VEGF, VEGFR1 and VEGFR2 (Santa Cruz Biotechnology, Santa Cruz, CA); COX-2 (Thermo Fisher Scientific, Fremont, CA); and mouse and rabbit IgG HRP-conjugated secondary antibody (Zymed Laboratories, San Francisco, CA). Other chemicals were obtained from Sigma-Aldrich (St. Louis, MO) unless specified. All reagents used for RT-PCR were purchased from Invitrogen (Carlsbad, CA).

### Western immunoblotting

Cells or tumor tissues were lysed in RIPA buffer containing 50 mM Tris, 150 mM NaCl, 0.1% SDS, 0.5% deoxycholate, 2 mM EDTA, 0.1% Triton X-100, 10% glycerol, 1 mM phenylmethylsulfonyl fluoride and 10 μg/ml aprotinin. The extracted protein was quantified using Coomassie® plus Protein Assay Reagent kit (Pierce, Rockford, IL). Total cellular proteins were separated by 8-15% SDS polyacrylamide gel electrophoresis. The proteins on the gel were transferred onto a nitrocellulose membrane (0.45 μm) and then probed with the respective primary antibodies, followed by the addition of the corresponding HRP-conjugated secondary antibodies. The immunoblots were visualized with enhanced chemiluminescence (ECL) reagents (Amersham Biosciences, Piscataway, NJ) and exposed to X-ray film. The membranes were then reprobed with β-actin for normalization. The band density on the film was analyzed by the Quantity One version 4.4.1 Basic Software (BioRad, Hercules, CA). In addition to representative bands, relative band densities (as arbitrary data) have also been illustrated in the form of histograms in order to better indicate the level of significance between treatment groups.

### Reverse transcriptase PCR (RT-PCR)

Cells were lysed in TRIzol® reagent to extract total cellular RNA. Single-stranded cDNA was synthesized from 5 μg of total RNA using M-MLV Reverse Transcriptase. The sequences of the primers used for PCR were: VEGF (NM_001025370, 226 bp) 5’-AAGGAGGAGGGCAGAATCAT-3’ (forward) and 5’-ATCTGCATGGTGATG TTGGA-3’ (reversed), HIF-1α (NM_001530, 193 bp) 5’-CCATTAGAAAGCAGT TCCGC-3’ (forward) and 5’-TGGGTAGGAGATGGAGATGC-3’ (reverse). GAPDH (595 bp) primers with sequence of 5’-CCACCCATGGCAAATTCCATGGCA-3’ (forward) and 5’-TCTAGACGGCAG GTCAGGTCCACC-3’ (reverse) were used as internal control. Conditions used for PCR consisted of 30 cycles for VEGF at 95°C for 2 min, 94°C for 30 s, 60°C for 30 s and 72°C for 30 s; 35 cycles for HIF-1α at 95°C for 15 min, 94°C for 45 s, 60°C for 45 s and 72°C for 45 s with a final incubation at 72°C for 10 min in a thermal cycler (GeneAmp PCR System 9700, Applied Biosystems). The RNA products were resolved by electrophoresis on 1.8% TAE agarose gel stained with ethidium bromide.

### Tumor xenograft in nude mice

The experimental procedures were approved by our institutional “Committee on the Use of Human and Animal Subjects in Teaching and Research (HASC)” with reference to the European Community guidelines for the use of experimental animals. Five-week-old female Balb/c-nu/nu mice were kept under sterile conditions in isolated pathogen-free ventilation chambers under ambient temperature of 20-22°C and 45-50% relative humidity. The animal rearing facility was maintained on a 12 h light–dark cycle. Mice were given free access to sterile food and water.

Cell suspension was prepared by trypsinization of confluent HCT 116 cells. Mice were randomly assigned to the control and AST treatment group. They were anesthetized with i.p. injection of 75 mg/kg ketamine and 10 mg/kg xylazine. Cell suspension was injected subcutaneously into the right thigh of the mice at the density of 2 × 10^6^ cells (in 100 μl DMEM medium). The day of tumor implantation was designated as Day 0. AST (100 mg/kg) was administrated orally once daily for 14 days after the tumors became palpable at Day 10. This dosage was acquired from our previous study as the optimal dose to alleviate tumor formation without causing systemic toxicity [5]. Tumor volume was measured using a digital caliper every 3 days and calculated as (length x width^2^)/2.

### Immunohistochemical analysis

Tumors were excised from HCT 116 xenograft mice on the day of sacrifice (Day 24). The paraffin-embedded samples were sectioned into 5 μm in thickness and mounted onto the slides. Slides were deparaffinized in xylene, followed by rehydration in ethanol. Antigens on the tumor tissues were retrieved by heating the sections with 10 mM sodium citrate, pH 6.0. Immunohistochemical analysis was performed by SuperPicture^TM^ 3^rd^ Gen Detection Kit (Invitrogen, Carlsbad, CA). The endogenous peroxidase activities were blocked by incubating with hydrogen peroxide. The sections were blocked with 2% bovine serum albumin (BSA) to minimize non-specific background and then incubated overnight with anti-COX-2 antibody at 1:200 dilutions (in 0.2% BSA) at 4°C. SuperPicture^TM^ reagent followed by 3,3-diaminobenzidine (DAB) reagent were incubated according to the manufacturer’s instruction. The sections were counterstained with Mayer’s hematoxylin. Negative control was performed with the same procedures without adding the primary antibody. All slides were evaluated by a minimum of two observers.

### Enzyme-linked immunosorbent assay (ELISA) for VEGF

To quantify levels of VEGF in the serum, blood samples were collected from HCT 116 xenografted nude mice on the day of sacrifice (Day 24) and allowed to clot at 4°C. Samples were centrifuged at 2,000 g for 10 min at 4°C. The serum was assayed by using a mouse ELISA kit for detecting VEGF (Calbiochem) according to the manufacturer’s instructions (Invitrogen, Carlsbad, CA).

### Statistical analysis

All data were presented as mean ± standard error of the mean (S.E.M.). Statistical significance of at least *p <* 0.05 was determined by one-way analysis of variance (ANOVA) followed by a post-hoc Least Significant Difference (LSD) test using the SPSS version 10.0 software.

## Results

### AST downregulated the protein expression of angiogenic factors bFGF and VEGF *in vitro*

**Figure 1:**
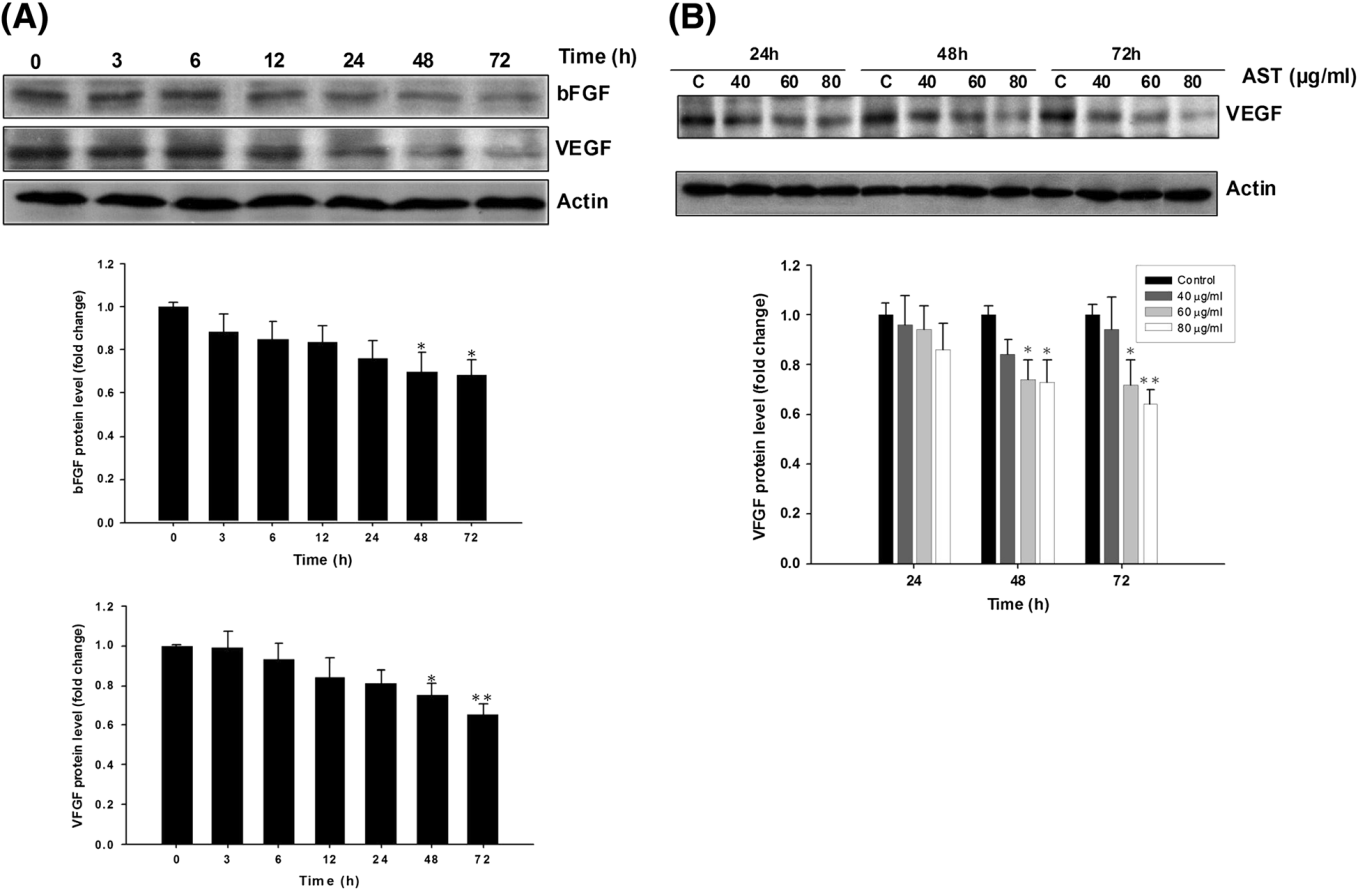
**AST downregulates pro-angiogenic factors in HCT 116 colon cancer cells.** (**A**) HCT 116 cells were treated with AST (80 μg/ml) and Western blotting was performed to analyze the level of bFGF and VEGF in a 72-hour time course study. (**B**) Various concentrations of AST (0, 40, 60 or 80 μg/ml) were administered to HCT 116 cells for 24, 48 or 72 h to determine its concentration-dependent effect on bFGF and VEGF level. Representative immunoblots from at least three independent experiments are shown. Relative band densities were summerized in histograms after normalization by β-actin. Arbitrary data are expressed as mean ± S.E.M., with statistical significance * p < 0.05, ** p < 0.01 when compared with the untreated group at 0 h (**A**) or the respective control group (**B**).

Basic fibroblast growth factor (bFGF) and VEGF are two key regulators for vasculogenesis secreted from the tumor cells and contribute synergistically to different stages of blood vessel formation [28]. Effect of AST on these two factors was evaluated in HCT 116 colon cancer cells. Our results show that AST significantly reduced the protein expression of both bFGF and VEGF in a time-dependent manner (Figure 1A). VEGF protein expression was also downregulated dose-dependently after AST treatment (Figure 1B). These results indicate that AST could attenuate the tumor progression through inhibition of intrinsic angiogenic factors.

### Effect of AST on PI3K/Akt/mTOR pathway and MAPK signaling

**Figure 2:**
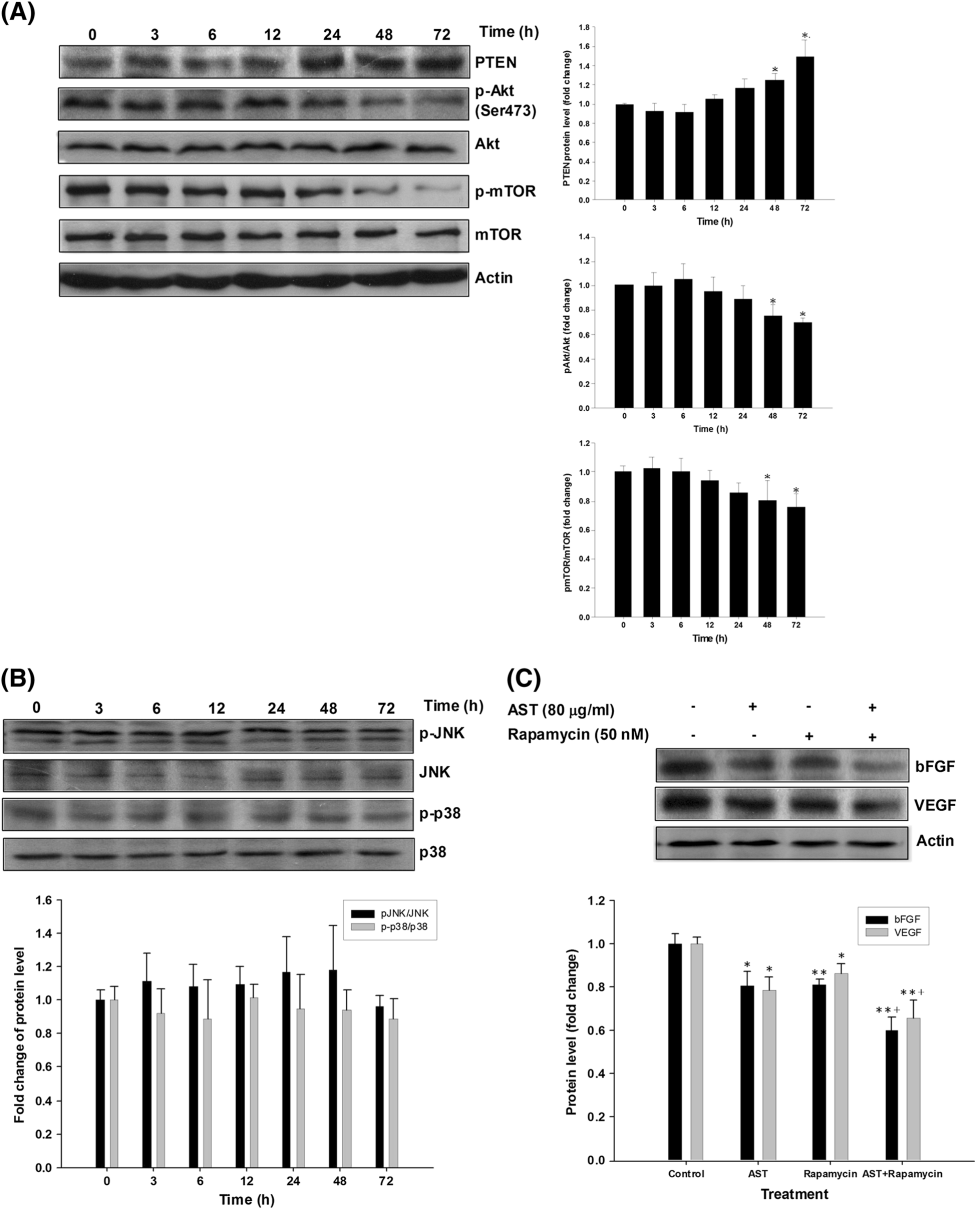
**AST acts by modulation of PI3K/Akt/mTOR signaling but does not involve the MAPK pathway.** HCT 116 cells were treated with AST (80 μg/ml) and Western blotting was performed to analyze the level of (**A**) PTEN, p-Akt, Akt, p-mTOR, mTOR and (**B**) p-JNK, JNK, p-p38 and p38 in a 72-hour time course study. (**C**) Combined effects of AST and rapamycin on the pro-angiogenic factors bFGF and VEGF. HCT 116 cells were incubated with AST (80 μg/ml) either in the absence or presence of the mTOR inhibitor rapamycin for 72 h. Rapamycin (50 nM) was added 30 min prior to the AST treatment. Representative immunoblots from at least three independent experiments are shown. β-actin was used as internal control in immunoblot assay. Arbitrary data are expressed as mean ± S.E.M., with statistical significance * p < 0.05, ** p < 0.01 when compared with the untreated group at 0 h (**A**) or control group (**B**); ^+^ p < 0.05 when compared with rapamycin group.

PI3K/Akt/mTOR pathway regulates various cellular activities. Its activation is associated with tumor progression including promotion of angiogenesis and invasion, and enhancement of metastatic potential in several human cancers, including colon cancer [29,30]. Therefore, several important signal transduction molecules in this pathway were examined in the present study, in which it is well known that PTEN serves as negative regulator in the PI3K/Akt pathway. Our time-course study shows that the protein expression of PTEN increased after 24 h of AST incubation. Activation of Akt was also studied by the assessment of phosphorylated form of Akt (p-Akt) at Ser 473. Results showed that p-Akt was downregulated after 48 h of AST treatment while no significant change in total Akt protein was observed. The effect of AST on mTOR which is a downstream target of Akt, was then investigated. AST could downregulate the phosphorylated form of mTOR (p-mTOR) upon 48 h of drug treatment without significant change in total mTOR level (Figure 2A).

The effect of AST on MAP kinase signaling, which is another known upstream pathway attributed to tumor angiogenesis and invasion [30,31] was also examined. Results show that AST did not cause significant change on the protein expression of either phosphorylated or total JNK as well as p38 over the course of study (Figure 2B). Thus, it appears that AST mainly exerts its anti-carcinogeneic effect by modulating Akt/mTOR signaling but not JNK or p38 MAP kinase signaling.

### Inhibitory effects of the combined treatment of AST and the inhibitors of PI3K/Akt/mTOR signaling on bFGF and VEGF level

To further investigate the involvement of PI3K/Akt/mTOR signaling in the AST-induced anti-angiogenic effects against colon cancer, HCT 116 cells were treated with PI3K inhibitor LY294002 (50 μM), Akt inhibitor (10 μM) or mTOR inhibitor rapamycin (50 nM), respectively. Results show that the protein expression of bFGF and VEGF was further reduced after co-treatment of AST and rapamycin for 72 h when compared to either AST or rapamycin treatment alone (Figure 2C). However, no further inhibition of these proteins expression could be observed in the combined treatment of AST with either LY294002 or Akt inhibitor (unpublished observations). Thus, the observed additive effect of AST and rapamycin suggests that the inhibitory action of AST on these angiogenic factors could at least partly act through mTOR signaling, but not the entire PI3K/Akt/mTOR pathway. Further investigation would be carried out to find out if AST affects individual regulatory molecules in mTOR signaling.

### Study of HIF-1α and VEGF expression under CoCl_2_-mimicked hypoxic condition

**Figure 3:**
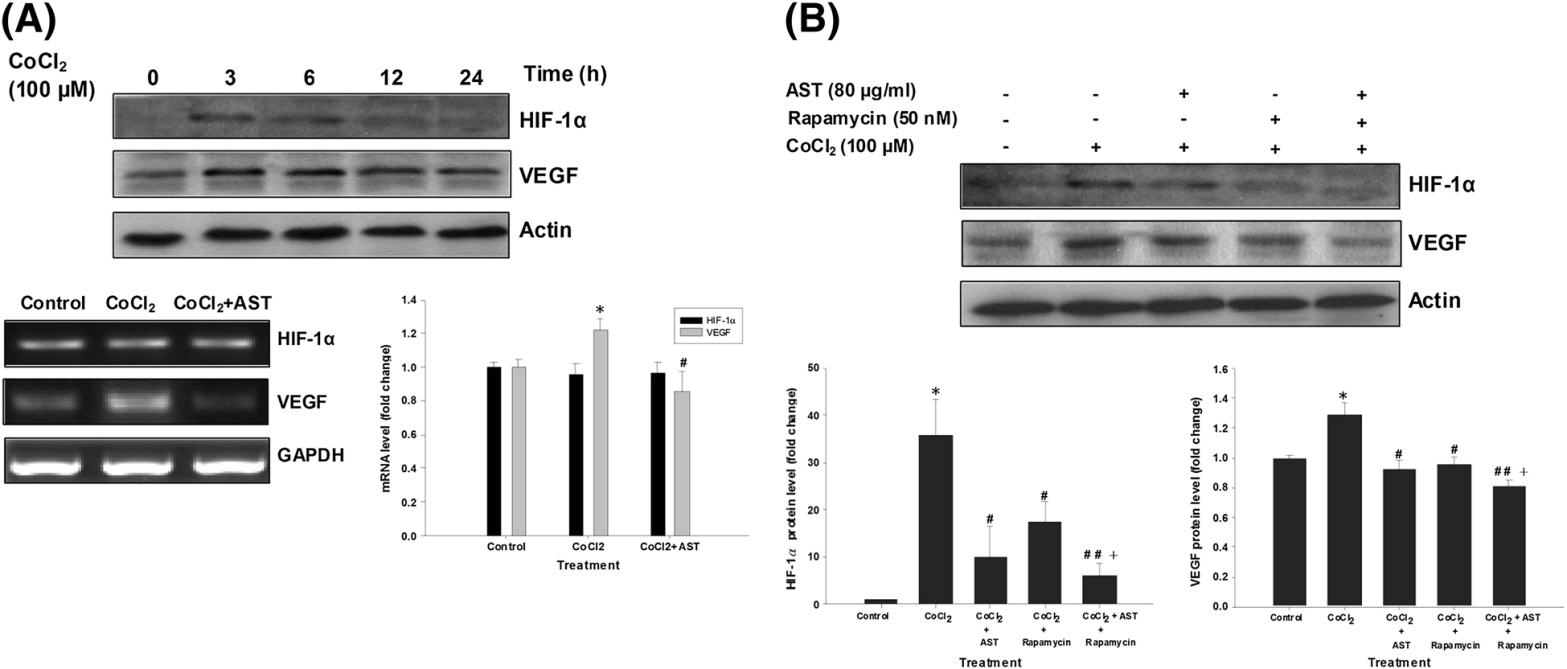
**AST suppresses HIF-1α and VEGF induction under hypoxia in colon cancer cells.** CoCl_2_ (100 μM) was added to the culture medium of HCT 116 cells 30 min prior to various drug treatments to mimic hypoxic condition. (**A**) The induction of HIF-1α and its downstream molecule VEGF by CoCl_2_ over a 24-hour course was monitored in HCT 116 cells. The effect of AST (80 μg/ml) on HIF-1α and VEGF protein and mRNA expression was then assessed. (**B**) Combined treatment of AST and rapamycin (50 nM) suppressed HIF-1α and VEGF protein expression under CoCl_2_-mimicked hypoxic condition in HCT 116 cells. Data represents the mean of at least three independent experiments. β-actin and GAPDH were used as internal control in immunoblot assay and RT-PCR, respectively. Arbitrary data are expressed as mean ± S.E.M., with statistical significance * p < 0.05 when compared with control group; ^#^ p < 0.05, ^##^ p < 0.01 when compared with CoCl_2_ hypoxia group; ^+^ p < 0.05 when compared with CoCl_2_ + rapamycin group.

The state of hypoxia is physically existed in tumor mass, which in turn stimulate angiogenesis to nourish the cells. Cobalt chloride (II) (100 μM) was added to HCT 116 cells to mimic hypoxic condition. HIF-1α protein actively undergoes degradation and is almost undetectable in HCT 116 cells under normoxic condition. Upon the exposure of CoCl_2_, the protein level of HIF-1α was elevated in HCT 116 cells and this accumulation of HIF-1α would transcriptionally upregulate VEGF mRNA level. CoCl_2_ does not induce HIF-1α mRNA expression. HIF-1α protein is continuously degraded under normoxic conditions by the ubiquitin-proteasome pathway, which is catalyzed by HIF prolyl hydroxylases. The hydroxylated HIF-1α is then recognized by a von Hippel-Lindau protein (pVHL). Cobalt mimics hypoxia by inactivating the hydroxylase activity and preventing the interaction between HIF-α and pVHL, thereby preventing the degradation of HIF-1α [32]. Our results show that CoCl_2_ did not affect the mRNA expression of HIF-1α but intensely induce the protein expression in HCT 116 cells, which is consistent with Fukuda’s findings using the same cell line [33]. AST treatment alone for 3–6 hours did not alter the protein expression of VEGF, HIF-1α and COX-2 (unpublished observations). Our results show that CoCl_2_-induced VEGF mRNA expression was significantly reduced to control level after AST treatment (Figure 3A). Furthermore, the induced protein expression of both HIF-1α and VEGF was abolished in AST-treated cells. We next investigated whether mTOR is involved in such suppression under the mimetic hypoxic condition. Cells were pre-treated with rapamycin for 30 min prior to the addition of CoCl_2_. Rapamycin (50 nM) could reduce the protein expression of HIF-1α and VEGF at 6 h while co-treatment of AST (80 μg/ml) showed an additional effect on such drop (Figure 3B). Based on the above results, it is probable that AST could act through mTOR signaling to regulate HIF-1α and VEGF under CoCl_2_-induced hypoxia.

### Effects of AST on the inhibition of COX-2 in colon cancer cells

**Figure 4:**
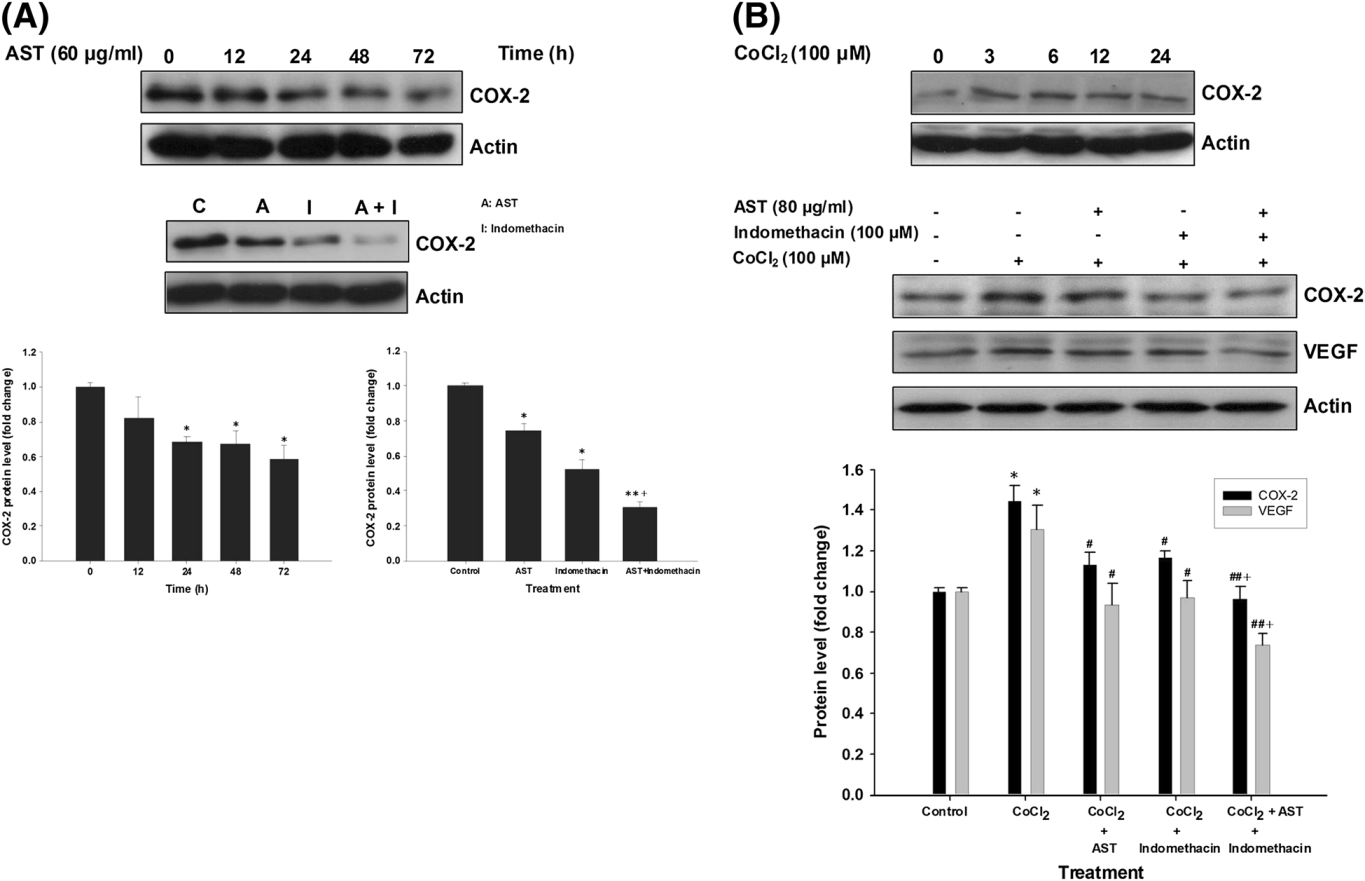
**AST suppresses COX-2 and VEGF induction under normoxia and hypoxia in colon cancer cells.** (**A**) Effects of AST alone {**A**} (60 μg/ml) or in combined treatment with indomethacin {A + I} on the level of COX-2 in HT-29 cells under normoxia. (**B**) Combined treatment of AST (80 μg/ml) and indomethacin (100 μM) suppressed the induced COX-2 and VEGF level under CoCl_2_-mimicked hypoxic condition in HCT 116 cells. Data represents the mean of at least three independent experiments. β-actin was used as internal control in immunoblot assay. Arbitrary data are expressed as mean ± S.E.M., with statistical significance * p < 0.05, ** p < 0.01 when compared with the untreated group at 0 h or control group; ^#^ p < 0.05, ^##^ p < 0.01 when compared with CoCl_2_ hypoxia group; ^+^ p < 0.05 when compared with indomethacin (A) or CoCl_2_ + indomethacin (**B**) group.

Blockade of COX-2 can suppress intestinal polyp formation and prevent colon carcinogenesis [34]. The role of COX-2 in tumor regulation and angiogenesis has been extensively investigated [17,20,35]. In this part of the study, HT-29 cells were used instead of HCT 116 cells because COX-2 is not expressed in the latter cell type under normoxic condition [18,36]. Our results show that AST (60 μg/ml) significantly reduced the protein expression of COX-2 in HT-29 colon cancer cells in a time-dependent manner under normoxia. The cells were treated with COX-2 inhibitor indomethacin (100 μM) with or without the combined treatment with AST. Combined treatment imposed greater inhibition on the protein expression of COX-2 when compared to individual drug effects (Figure 4A).

COX-2 was reported to be induced by hypoxia in colon cancer and was transcriptionally regulated by HIF-1α [18]. Therefore, the effect of AST on COX-2 inhibition under hypoxic condition had been studied. COX-2 protein expression can be induced in HCT 116 cells under the condition of CoCl_2_-mimicked hypoxia. Our results demonstrate that either AST or indomethacin (100 μM) alone could suppress CoCl_2_-induced COX-2 and VEGF protein expression in HCT 116 cells. Further downregulation of these parameters was observed with combined treatment of indomethacin and AST (Figure 4B). Taken together, we propose that AST could downregulate VEGF level through inhibition of COX-2 under both normoxic and hypoxic conditions.

### AST reduced tumor volume in HCT 116-xenografted nude mice

**Figure 5:**
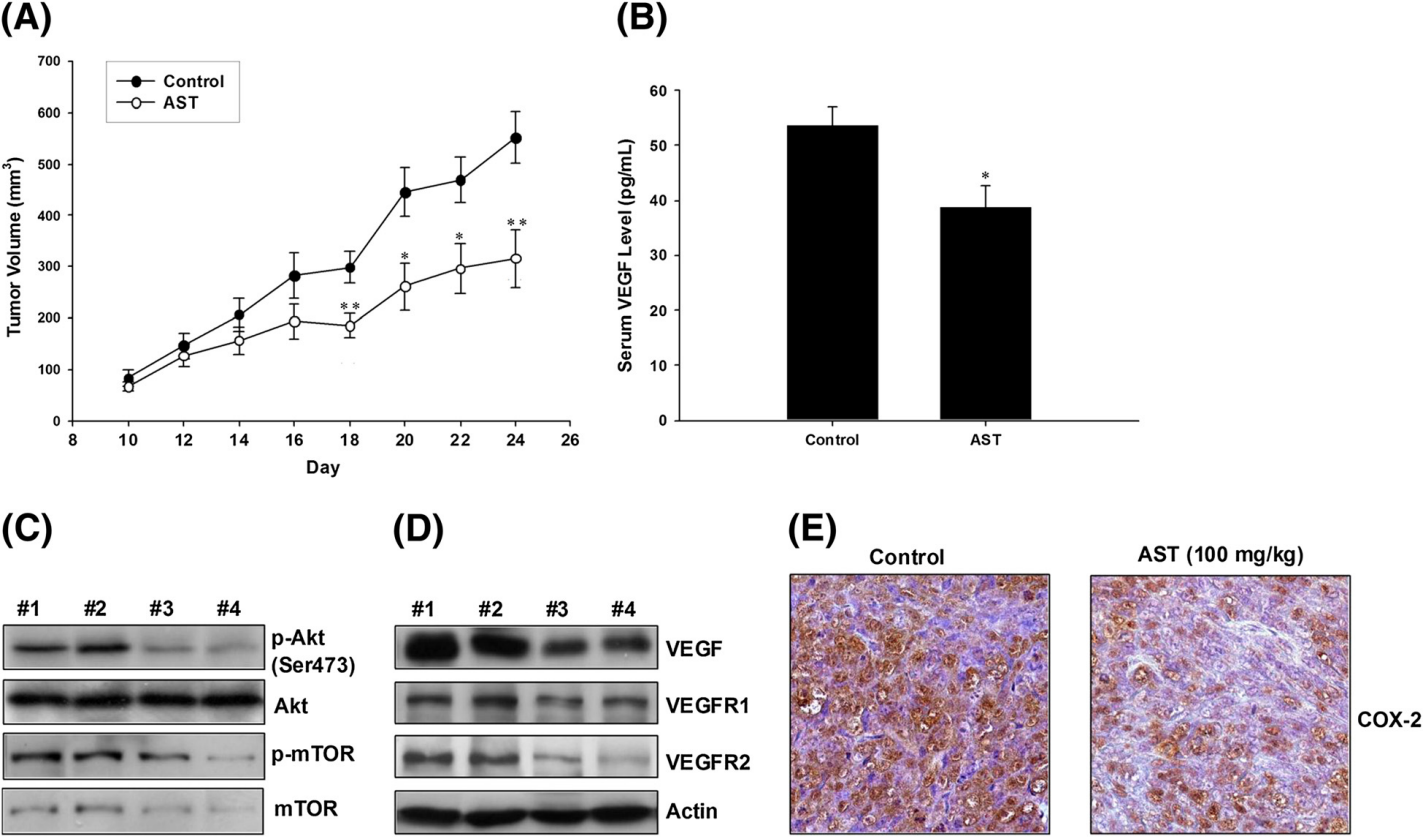
**AST inhibits tumor growth and suppresses tissue VEGF level by modulating Akt/mTOR signaling and COX-2.** HCT 116 colon cancer cells (2 × 10^6^) were injected subcutaneously to the Balb/c nu/nu mice. AST (100 mg/kg) was administrated orally once daily for 14 days after the tumors became palpable at Day 10. Tumors from the xenografted mice were excised at the day of sacrifice. (**A**) Tumor volume and (**B**) serum VEGF level in the control and AST-treated groups of animals were measured. Data are expressed as mean ± S.E.M. (n = 6), with statistical significance * p < 0.05, ** p < 0.01 when compared with control group. (**C**) Tissue level of p-Akt, Akt, p-mTOR, mTOR, and (**D**) the pro-angiogenic factors VEGF, VEGFR1 and VEGFR2 was examined in untreated control (#1-2) and AST-treated (#3-4) groups of animals. Representative immunoblots from tumor tissues obtained from six animals in each group are shown. β-actin was used as internal control in immunoblot assay. (**E**) Histological examination of COX-2 level in tissue sections of HCT 116 xenografted nude mice with or without AST treatment was performed. Representative microphotographs of control and drug-treated groups are shown (200x magnification).

Mice were orally fed with saline or AST (100 mg/kg) once daily for 14 consecutive days. No significant change in body weight and no mortality were observed in all treatment groups throughout the entire course of the experiment. Tumor volume was reduced by 42.6% (552 ± 49.6 mm^3^ in control versus 317 ± 56.0 mm^3^ in AST-treated mice after 14-day drug treatment compared to saline control (Figure 5A).

### AST reduced serum VEGF level in tumor xenograft nude mice

Angiogenesis is sometimes linked with advanced cancers, while blockade of VEGF production significantly diminishes the tumor volume *in vivo*[8]. The effect of AST on VEGF production was therefore investigated by measurement of the circulating level of VEGF in tumor-bearing mice. Treatment with AST resulted in a significant reduction in serum VEGF level (Figure 5B), providing an insight of its anti-tumor effect at least partly due to the inhibition of angiogenesis.

### AST attenuated angiogenesis in nude mice xenograft through inhibition of mTOR and COX-2

In HCT 116 xenografted nude mice model, tumors were excised and Western blotting was performed in tumor tissues. Our results show that the protein expression of VEGF and its receptors VEGFR1 and VEGFR2 was downregulated in AST-treated mice compared to that of control (Figure 5D). Similarly, p-Akt and p-mTOR level was also decreased after AST treatment (Figure 5C). Moreover, AST could inhibit COX-2 expression in the tumor section from nude mice xenograft (Figure 5E). All these findings re-confirm the *in vitro* results about the inhibitory effects of AST on tumor angiogenesis that are regulated by modulation of both mTOR and COX-2 signaling in colon cancer cells.

## Discussion

Angiogenesis is essential for tumor progression because tumor mass cannot grow bigger than 2 mm^3^ without the nourishment of blood vessels. Moreover, vascularization is required for the process of extravasation in metastasis [7]. Hence, establishment of chemotherapeutic strategy by blocking angiogenesis attracts much attention in recent years. Besides, alteration of the cellular adaptation to hypoxia is also fundamental in cancer treatment because angiogenesis or other metabolic modifications will be stimulated to maintain tumor cell survival [37]. Our ongoing study revealed that AST could significantly reduce tumor growth in nude mice by inhibiting cell proliferation and promoting apoptosis. We then hypothesized that AST may also attenuate tumor angiogenesis both *in vitro* and *in vivo*. VEGF is the most potent pro-angiogenic factor to facilitate tumor angiogenesis. Blockade of VEGF production significantly inhibited tumor growth in various mice models [8-10]. In the present study, we have demonstrated that AST could reduce tumor volume in HCT 116 nude mice xenograft with a concomitant decrease in serum VEGF level, suggesting that AST exerts its anti-tumor effect in colon cancer by targeting VEGF and its associated signaling pathways.

PI3K/Akt kinase cascade is one of the most important signaling pathways in cancer development, which correlates strongly with angiogenesis, invasiveness and metastasis of the tumor. Our results have shown that AST significantly reduced protein expression of the angiogenic factors bFGF and VEGF. We recently reported that AST exhibited antitumorigenic and proapoptotic actions in HCT 116 cells through induction of a novel proapoptotic protein NAG-1, which could be intensified by the co-treatment of PI3K-Akt inhibitors [4]. Based on this background, the transcriptional factors involved in PI3K/Akt signaling were further examined in the present study. Data from our *in vitro* experiments show that AST caused upregulation of the tumor suppressor PTEN, inhibition of Akt phosphorylation at Ser 473 as well as downregulation of p-mTOR. These findings implicate the possible involvement of PI3K/Akt/mTOR signaling in the anti-carcinogenic action of AST. To confirm whether such regulation is related to the anti-angiogenic effect, cells were treated with the combination of either LY294002, Akt inhibitor or rapamycin with AST. Neither LY294002 or Akt inhibitor alone reduced the level of bFGF and VEGF, while co-treatment with AST did not lead to additional effect (unpublished observations). In contrast, rapamycin alone induced significant downregulation of these growth factors, while a greater inhibitory effect was achieved with combined treatment of rapamycin and AST. Based on these observations, PI3K and Akt are less likely to be the target molecules of AST responsible for its downregulation of the two angiogenic factors. Alternatively, it has been known that activation of MAPK signaling pathway may also be involved in promoting tumor angiogenesis and invasion [30,31]. We have shown that AST could not cause significant activation of JNK and p38 MAP kinase. Although our previous study have indicated that ERK1/2 activity was increased within 6 h following AST treatment, and that this transient induction could be related to its proapoptotic function involving Egr-1 induction [5,38], no evidence has indicated a direct correlation with angiogenesis in this study. From the above information, we propose that AST could suppress the angiogenic factors through inhibition of mTOR signaling rather than via modulation of the MAPK pathway.

VEGF is transcriptionally regulated by HIF-1α, resulting in overexpression in human colon cancer biopsies. Moreover, forced induction of HIF-1α in HCT 116 cells increases tumor growth and angiogenesis in nude mice [39]. Physiological stimulus such as hypoxia can inhibit the degradation of the HIF-1α subunit, and then the accumulation of HIF-1α dimerizes with the constitutively expressed HIF-1β subunit and activates the transcription and stabilization of VEGF gene [40]. Stabilization of HIF-1α can be also induced by transition metals such as cobalt, nickel, and manganese, by iron chelators and by antioxidants. These metallic ions mimic hypoxia by binding to the heme portion of oxygen-sensing molecules inside the cells [41]. CoCl_2_, being a common hypoxic-mimetic agent, was used to investigate the effect of AST on the expression of HIF-1α and VEGF under hypoxic condition. CoCl_2_ did not affect the mRNA expression of HIF-1α but intensely induce HIF-1α protein expression in HCT 116 cells. AST could significantly reduce the CoCl_2_-induced HIF-1α protein expression and VEGF mRNA expression under the mimetic hypoxic condition. At present, it remains unclear whether it is the degradation rate or the protein synthesis of HIF-1α that has been reduced by AST [33,40], which requires further investigation. Following this, the effect of combined rapamycin and AST treatment under the mimetic hypoxic condition was also investigated. An even greater downregulation of HIF-1α and VEGF expression was achieved in the combined treatment when compared to the effects of either agent alone. mTOR was found to be an upstream activator of HIF-1α expression in cancer cells [25]. Direct interaction between these two proteins may exist since there has been evidence showing that HIF-1α activity during hypoxia requires an mTOR signaling motif located at the N terminus of HIF-1α, while high level of HIF activity can be reversed by rapamycin [42]. Based on the above interrelationship, we suggest that AST could suppress tumor angiogenesis by interfering with the mTOR/HIF-1α axis under CoCl_2_-stimulated hypoxia.

COX-2 expression has been found to be upregulated in most human tumors including colon, lung, breast and prostate, which also attributes to cancer-related inflammation, tumor cell proliferation, resistance to apoptosis and angiogenesis [43]. Evidence has shown that HIF-1 directly binds to a specific HRE on the COX-2 promoter in such a way that COX-2 serves as a HIF-1 target gene and is induced during hypoxia [18]. The same report also mentions that COX-1 expression would remain unchanged in colon cancer cells under hypoxia, suggesting the significance of the COX-2 isozyme alone in the regulation of the hypoxia-adaptive response [44]. As mentioned earlier, AST could downregulate VEGF expression through mTOR signaling. Nonetheless, since the expression of VEGF was not completely inhibited by rapamycin in HCT 116 cells, other signaling pathways may also be involved in the regulation of VEGF, including the COX-2 cascade. Under hypoxia, COX-2 can trigger VEGF induction and the response is independent of the COX-2 cellular level [45]. HCT 116 cells are natively COX-2-negative, whereas another colon cancer cell line HT-29 has high constitutive COX-2 level. Thus, HT-29 cells had been chosen to determine the effect of AST on COX-2 expression under normoxic condition. AST significantly decreased constitutive protein expression of COX-2 in HT-29 cells. On the other hand, COX-2 expression could be eminently induced in COX-2-deficient HCT 116 cells by CoCl_2_. We found that protein expression of both COX-2 and VEGF would be reduced following 24 h of AST treatment, with further decrease when indomethacin was co-treated. This implicates that AST could have inhibited VEGF expression through downregulation of COX-2 under hypoxia. Further investigation could be established to explore the direct effect of AST on the generation of COX-2 products such as prostaglandins and thromboxanes, since their increased production has been reported to induce VEGF secretion from tumor cells [46].

In the HCT 116-xenografted nude mice model, daily intake of AST markedly reduced the tumor volume of the mice when compared to the control animals. Results show that the decrease of VEGF expression in tumor tissue of AST-treated mice was correlated with the downregulation of p-Akt, p-mTOR and COX-2, which are consistent with the *in vitro* findings. VEGFR-1 and VEGFR-2 are receptors distinctively present on vascular endothelial cells, which have strong association with cell proliferation, migration and induction of vascular permeability [47]. VEGFR-1 was found in colorectal cell lines including HCT 116 to promote cell migration and invasion [48]. Thus, the decreased expression of both VEGFR-1 and VEGFR-2 in tumor tissues suggests that AST could attenuate tumor progression via inhibition of angiogenesis and metastasis [49]. As a matter of fact, there is a close relationship between matrix metalloproteinases (MMPs) and VEGF in tumor progression, of which MMP-9 induces the release of biologically active VEGF in the culture of ovarian tumor cells and in ascites of ovarian tumor-bearing mice [50]. MMPs are secreted by recruited macrcophages, mast cells and fibroblasts, of which one important role of MMPs is to degrade and remodel the surrounding extracellular matrix (ECM) by proteolysis. When ECM is degraded, angiogenic factors being sequestered there in addition to cytokines and tumor cells will be released together, further facilitating the growth and infiltration of new blood vessels into the tumor [51]. We have previously discovered that AST could downregulate MMPs and other factors associated with ECM degradation together with its modulation of tissue VEGF level (unpublished observations). The correlated interactions between VEGF, MMPs and other ECM-related proteases such as urokinase plasminogen activator (uPA) as well as their potential regulation by AST during control of tumor progression still needs to be elucidated in the near future.

## Conclusions

To summerize, we conclude that AST is able to attenuate tumor growth by downregulation of VEGF production, which involves modulation of mTOR and COX-2 signaling. The inhibition of COX-2 may also ameliorate the inflammatory responses that could be associated with further cancer development [23]. In the present study, AST was found to have multiple molecular targets, while its mode of action involves robust synergistic interaction with other novel tumor markers and modulators as implicated from our previous works [4,5]. All these evidences suggest that AST is a potential candidate for further development as an adjuvant in modern chemotherapeutic treatment of colon cancer, perhaps to work together with other effective agents such as bevacizumab to further boost the anti-cancer effects of each other.

## Abbreviations

AST: Total Astragalus Saponins; bFGF: Basic Fibroblast Growth Factor; CoCl_2_: Cobalt-chloride (II); COX-2: Cyclooxygenase-2; HRE: Hypoxia Responsive Element; HIF-1: Hypoxia-inducible Factor-1; mTOR: Mammalian target of rapamycin; PGs: Prostaglandins.

## Competing interests

The authors declare that they have no competing interests.

## Authors’ contributions

JKK formulated the original ideas and working hypothesis (PI of the research grant); JKK and KKA designed the research studies; KKA, PL and LC carried out the experiments; LC and KKA analyzed and interpreted the data; KKA and LC wrote the first draft of the manuscript; JKK revised and wrote the final draft of the manuscript. All authors read and approved the final manuscript.

## Pre-publication history

The pre-publication history for this paper can be accessed here:

http://www.biomedcentral.com/1472-6882/12/160/prepub
